# 233 Flexibility matters. Psychological flexibility fully mediates the effect of regular physical activity on life satisfaction

**DOI:** 10.1093/eurpub/ckae114.068

**Published:** 2024-09-26

**Authors:** Lukasz Krzywoszanski, Wiktor Potoczny, Radoslawa Herzog-Krzywoszanska

**Affiliations:** University Of The National Education Commission, Krakow, Poland; University Of The National Education Commission, Krakow, Poland; University Of The National Education Commission, Krakow, Poland

## Abstract

**Purpose:**

Regular physical activity is essential for maintaining quality of life, good health and managing chronic diseases. It has positive effects on physical, mental and social well-being. However, the mechanisms responsible for this effect are still not fully understood. Psychological flexibility is the ability to adapt to fluctuating situational demands and to reframe mental perspectives in ways that facilitate the pursuit of meaningful life goals. Cognitive defusion (as opposed to cognitive fusion) and willingness to engage in committed action are important aspects of psychological flexibility. Current research aims to explore the possible mediating role of cognitive defusion and committed action in the relationship between regular exercise and mental well-being.

**Methods:**

186 adults from the general population took part in the online survey and reported how much physical activity they do on a regular basis. They also completed Satisfaction with Life Scale, Cognitive Fuzion Questionnaire and Committed Action Questionnaire. Participants’ self-reports of their average weekly physical activity over the previous year were used to classify them into four levels of physical activity, as defined by the World Health Organisation’s guidelines on physical activity and sedentary behaviour. The direct and indirect effects of physical activity levels on life satisfaction, with scores on the Cognitive Fuzion Questionnaire and the Committed Action Questionnaire as mediating variables, were assessed in a parallel mediation model.

**Results:**

The parameter estimates for both indirect paths were significant, whereas for the direct path it was not significantly different from zero. This indicates that cognitive fusion and willingness to engage in committed action fully mediated the relationship between the levels of physical activity and life satisfaction. Parameter estimates for both components of the indirect pathway leading through scores on the Committed Action Questionnaire were positive, and for both components of the indirect pathway leading through scores on the Cognitive Fuzion Questionnaire were negative.

**Conclusions:**

The results suggest that involvement in regular physical activity may contribute to more flexible psychological functioning, which in turn leads to greater life satisfaction. They shed new light on the mechanisms responsible for the beneficial effects of regular physical activity on psychological well-being and mental health.

